# Formulation and Comparative Characterization of SLNs and NLCs for Targeted Co-Delivery of Paclitaxel and Hydroxytyrosol Carboxylic Acid Esters Against Triple-Negative Breast Cancer

**DOI:** 10.3390/pharmaceutics17091208

**Published:** 2025-09-16

**Authors:** Elena Peira, Simona Sapino, Daniela Chirio, Fabio Bucciol, Flavia Turku, Emanuela Calcio Gaudino, Giancarlo Cravotto, Chiara Riganti, Marina Gallarate

**Affiliations:** 1 Department of Drug Science and Technology, University of Turin, Via Pietro Giuria 9, 10125 Turin, Italy; simona.sapino@unito.it (S.S.); daniela.chirio@unito.it (D.C.); fabio.bucciol@unito.it (F.B.); flavia.turku@unito.it (F.T.); emanuela.calcio@unito.it (E.C.G.); giancarlo.cravotto@unito.it (G.C.); 2 Department of Oncology, University of Torino, Via Santena 5/bis, 10126 Turin, Italy; chiara.riganti@unito.it

**Keywords:** solid lipid nanoparticles, nanostructured lipid carriers, triple-negative breast cancer, hydroxytyrosolcarboxylic acid esters

## Abstract

**Background:** The management of triple-negative breast cancer (TNBC) remains a therapeutic challenge due to the presence of multidrug resistance (MDR) and hypoxia-induced chemoresistance, both of which substantially reduce the efficacy of conventional chemotherapy. Although certain natural compounds have shown the ability to modulate these resistance mechanisms, their clinical application is hindered by poor solubility and limited bioavailability. Among such phenolic compounds, 7-hydroxytyrosol (HTyr)—a phenolic compound from olive oil and olive leaves—has been reported to modulate hypoxia-inducible factor-1 (HIF-1). **Methods:** In this study, we developed hyaluronic acid (HA)-decorated solid lipid nanoparticles (SLNs) and nanostructured lipid carriers (NLCs) for the targeted and synergistic co-delivery of paclitaxel (PTX) and hydroxytyrosol carboxylic acid esters (C_n_-HTyrCA), precursors that share the antioxidant biphenolic moiety with HTyr. **Results:** Among the formulations tested, SLNs of trilaurin (TL) exhibited the highest entrapment efficiency (EE%), optimal average particle size, Zeta potential, and good colloidal stability. Of the synthesized C_n_-HTyrCA derivatives, C_8_- and C_10_-HTyrCA showed superior loading capacity. In vitro release profiles indicated a sustained drug release pattern for both nanoparticles. HA decoration led to a marked increase in particle size and induced a shift in surface charge, confirming successful decoration and suggesting enhanced targeting potential via HA-CD44 interaction. Cytotoxicity assays conducted on MDA-MB-231 cells showed that PTX-loaded TL-SLNs exerted enhanced antitumor activity, particularly when HA-decorated, and a synergistic effect was observed upon co-administration with SLNs loaded with C_8_-HTyrCA. **Conclusions:** Overall, our findings support the potential of SLN as a promising strategy to overcome key resistance mechanisms in TNBC, enabling reduced chemotherapeutic dosing and improving therapeutic outcomes.

## 1. Introduction

Triple-negative breast cancer (TNBC) is a subtype of breast cancer accounting for about 10–20% of diagnosed cases. It is characterized by the lack of expression of both estrogen and progesterone receptors and the human epidermal growth factor receptor 2 (HER 2) [1].

Due to this receptor-negative profile, patients affected by TNBC generally face an unfavorable prognosis and cannot benefit from either endocrine-based therapies or HER2-targeted treatments [2]. Overall, TNBC is associated with poorer clinical outcomes compared with non-TNBC tumors, a phenomenon often referred to as the “triple-negative paradox” [3]. This subtype is further linked to higher recurrence rates and a markedly reduced survival time.

Although novel targeted strategies continue to be investigated, the current first-line therapeutic approach for TNBC remains primarily based on surgery in combination with chemotherapy. However, these treatments typically result in lower progression-free and overall survival rates [4]. Furthermore, drugs commonly employed in metastatic breast cancer are largely ineffective in TNBC, as their mechanisms of action are directed against receptors not expressed in this tumor type. The scarcity of specific targeted options for TNBC, together with the emergence of multi-drug resistance (MDR), represents a major limitation to effective chemotherapy [5].

MDR has been recognized as a crucial mechanism underlying therapeutic resistance in TNBC. It is predominantly associated with the overexpression of ATP-binding cassette (ABC) efflux transporters, among which P-glycoprotein (P-gp) is the most extensively studied due to its broad substrate specificity [6]. Elevated levels of P-gp have been directly implicated in resistance to several classes of chemotherapeutic agents, including taxanes, anthracyclines, vinca alkaloids, and epipodophyllotoxins [7].

Given the considerations outlined above, and considering the significant contribution of TNBC to breast cancer-related mortality, the identification of new and effective therapeutic strategies is urgent. Since the P-gp actively mediates drug efflux in MDR-TNBC cells, many studies have focused on the development of P-gp inhibitors to enhance chemosensitivity [8,9].

Another critical factor contributing to therapeutic failure is hypoxia, a condition defined by oxygen tension levels below 2.5 mmHg, which occurs in approximately 60% of solid human tumors and markedly decreases the responsiveness of cancer cells to chemotherapy [10,11]. In experimental models, hypoxic conditions have consistently been shown to promote resistance to anticancer agents in different cell lines [12]. A key regulator of cellular adaptation to hypoxia is Hypoxia-Inducible Factor 1 (HIF-1), a heterodimeric composed of an oxygen-sensitive α-subunit and a constitutively expressed β-subunit. Once translated via the PI3K/Akt/mTOR signaling pathway, HIF-1α causes an upregulation of survival-promoting proteins and increases the aggressiveness of hypoxic tumor cells.

Importantly, the induction and overexpression of HIF-1 are strongly correlated with an increased P-gp expression, thereby facilitating MDR development and worsening clinical prognosis [13].

In light of these mechanisms, the search for pharmacologically safe P-gp and/or HIF-1 inhibitors has increasingly turned toward natural compounds [14,15]. The long-standing use of herbal medicines provides a strong rationale for their favorable safety profile [16]. Indeed, several studies worldwide have demonstrated that multiple classes of phytochemicals—including flavonoids, stilbenes, coumarins, alkaloids, terpenoids, and saponins—are capable of sensitizing MDR cancer cells to chemotherapy [17]. Moreover, the consumption of olive oil is associated with a lower incidence of breast cancer. Hydroxytyrosol (HTyr), also known as 3,4-dihydroxyphenylethanol, belongs to the family of the *ortho*-diphenolic compounds. These compounds are present in small amounts in the soluble fraction of extra virgin olive oil. HTyr is abundantly found in the leaves of the olive tree (*Olea europaea* L.) and originates from the hydrolysis of oleuropein (a phenolic bitter compound found in green olive skin). Notably, its concentration increases during the ripening of olives and the storage of olive oil. Moreover, recent studies have highlighted the efficacy of innovative extraction technologies, such as ultrasound-assisted extraction and pulsed electric fields, in enhancing the extraction yield of natural antioxidants—including HTyr—in extra virgin olive oil, without altering its quality characteristics. These approaches represent promising strategies to improve the nutritional and functional value of oil [18].

It is known that the hypoxic microenvironment in solid tumors plays a crucial role in cancer progression and in the failure of cancer treatments. Since HTyr is one of the major bioactive compounds in olive oil, some Authors [19] delved into its modulatory role on HIF-1, showing that HTyr decreases HIF-1α protein in MCF-7 breast cancer cells, probably by downregulating oxidative stress and inhibiting the PI3K/Akt/mTOR signaling pathway.

The co-administration of chemotherapeutic agents and HIF-1 modulators faces pharmacokinetic challenges, including differences in permeability, bioavailability, and metabolism. Lipid-based nanosystems, such as solid lipid nanoparticles (SLNs) and nanostructured lipid carriers (NLCs), have been proposed to improve drug delivery and therapeutic efficacy.

In a previous study [20], we showed that curcumin (CUR)-loaded SLNs enhanced doxorubicin (DOXO) efficacy by 5–10 fold in P-gp–expressing TNBC cells by reducing intracellular reactive oxygen species (ROS), inhibiting the Akt/IKKα-β/NF-κB pathway, and downregulating P-gp promoter activation. This suggests that the combined use of CUR-loaded SLNs with DOXO represents a promising and well-tolerated strategy to counteract P-gp–mediated chemoresistance in TNBC.

In a subsequent paper [21], SLNs and NLCs, both unmodified and hyaluronic acid (HA)-decorated, were loaded with CUR, DOXO, and aminoflavone (AF), a modulator of HIF-1α currently under clinical investigation [22]. When tested on DOXO-sensitive and DOXO-resistant TNBC cell lines, HA-decorated SLNs promoted DOXO accumulation in P-gp–expressing cells and significantly reduced cell viability, with further enhancement upon co-incubation with CUR- and AF-loaded HA-decorated SLNs. These findings support the development of nanoparticle-based strategies to overcome resistance mechanisms in TNBC, and HA decoration suggests enhanced targeting potential via HA-CD44 interaction [23].

To improve the bioavailability and stability of antioxidant *ortho*-diphenolic compounds, lipophilic ethers or esters bearing long alkyl chains (C_10_−C_16_) have been extensively investigated for pharmaceutical applications [24], including their incorporation into various drug delivery systems. In particular, given the extensive literature on HTyr and its derivatives [25,26], we particularly focused on its carboxylated analog, hydroxytyrosol carboxylic acid (HTyrCA), chemically known as 2-(3,4-dihydroxyphenyl)acetic acid, which remains comparatively less explored. In fact, our strategy focused on finding a cost-effective alternative to pure HTyr by exploiting this structurally related compound. Indeed, both HTyr and HTyrCA share the 3,4-dihydroxyphenyl moiety, a well-established pharmacophore responsible for potent antioxidant activity. This catecholic structure is retained in HTyrCA, supporting the hypothesis that the compound may retain antioxidant potential comparable to HTyr. In fact, previous studies have confirmed the radical scavenging capacity of HTyrCA and its lipophilic esters, with some derivatives exhibiting antioxidant activity similar or even superior to HTyr, particularly in lipophilic environments relevant to pharmaceutical formulations [27,28].

With this in mind, we have attempted to develop a series of lipophilic HTyrCA esters by selectively esterifying the carboxyl group with long-chain alcohols. This structural modification serves multiple purposes: improving membrane permeability and bioavailability, facilitating efficient incorporation into lipid nanocarriers, and reducing production costs compared to synthetic HTyr derivatives.

Based on these assumptions, the aim of our research is to develop an innovative therapeutic strategy that utilizes HA-decorated lipid nanosystems, separately loaded with PTX and a series of lipophilic esters of hydroxytyrosol carboxylic acid (C_n_-HTyrCA), to improve antitumor efficacy and overcome chemoresistance mechanisms in TNBC.

## 2. Materials and Methods

### 2.1. Materials

Hyaluronic acid sodium salt (mol. wt. 8.000–15.000) and Compritol^®^888ATO (glyceryl dibehenate) were purchased from Farmalabor (Barletta, Italy), Precirol^®^ATO5 (glyceryl distearate) was provided as a kind gift sample from Gattefossè (Saint Priest, France), cetyltrimethylammonium bromide (CTAB), stearic acid, palmitic acid, taurocholic acid sodium salt hydrate (purity ≥ 95%) glyceryl monostearate (GMS), ethyl acetate (EA, purity ≥ 99.5%), propylene glycol (PG, purity ≥ 99.5%), myristic acid (purity ≥ 98.5%), sesame oil (refined), dimethyl sulfoxide (DMSO, purity ≥ 99.9%), benzyl alcohol, Pluronic^®^F-68, Polyvinyl alcohol 9000 (PVA9000) Sephadex^®^G-25, Sepharose^®^CL-B4 and Tween^®^80 were purchased from Merck (Darmstadt, Germany), Epikuron^®^200 (phosphatidylcholine 92%) was from Cargill (Minneapolis, MN, USA), Cremophor^®^RH60 (PEG-60 hydrogenated castor oil) from BASF (Ludwigshafen am Rhein, Germany) and trilaurin (TL, 98% min.) from Alfa-Aesar (Ward Hill, MA, USA). PTX was kindly provided by Indena S.p.A (Palestro, Italy). HTyrCA (3,4-dihydroxyphenylacetic acid, 98%) was purchased from Thermo Fisher Scientific (Fisher Scientific, Milan, Italy). The MilliQ water purification system was used to obtain deionized water (Millipore, Bedford, MA, USA). Other reagents were purchased from Merck, and all solvents were HPLC-gradient grade.

### 2.2. Synthesis and Characterization of Active Compounds

**Scheme 1:**
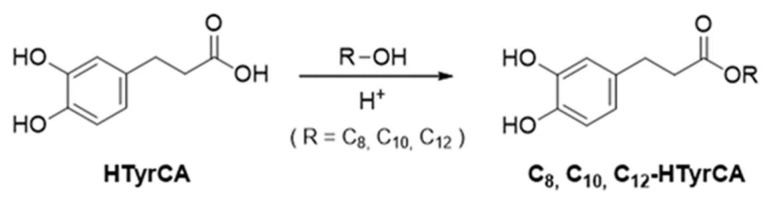
Synthesis of HTyrCA esters: C_8_-HTyrAC, C_10_-HTyrCA and C_12_-HTyrCA.

General synthetic procedure for the C_n_-HTyrCA was reported in Scheme 1: 500 mg of HTyrCA (2.98 mmol) were dissolved in 5 mL of 1-octanol (32 mmol) or 1-decanol (26 mmol) and 1-dodecanol (22 mmol), followed by the addition of 50 μL of H_2_SO_4_ (0.93 mmol). The reaction mixture was then stirred at 800 rpm and heated at 120 °C for 2 h in an Xelsius reactor (LabTech S.r.l., Sorisole, Italy). Upon completion of the reaction, the mixture was diluted with 10 mL of EA, and the unreacted acid was removed by liquid–liquid extraction with distilled water. The organic layer was collected, dried over anhydrous Na_2_SO_4_, and concentrated under reduced pressure using a rotary evaporator to remove EA. The excess alcohol was subsequently eliminated by vacuum distillation using a Liebig condenser.

The same compounds could also be synthesized more efficiently and quickly in a microwave-assisted autoclave (SynthWAVE from Milestone S.r.l., Sorisole, Italy). The synthesis was prepared as previously described, in 15 mL glass vials with internal mixing provided by magnetic stirrers. In this case, complete conversion was observed in just 1 h.

The purity of the resulting esters (C_8_-HTyrAC, C_10_-HTyrCA, and C_12_-HTyrCA) was first evaluated by GC–MS 6850 system, Megawax 5-MPS column (Agilent, Santa Clara, CA, USA) and further confirmed by HPLC analysis. In detail, for the GC-MS analysis, the oven temperature program was set as follows: initial temperature of 50 °C for 5 min, ramped to 100 °C at 10 °C/min for 1 min, ramped to 230 °C at 20 °C/min for 1 min, ramped to 300 °C at 20 °C/min for 5 min, and held for 15 min. The injector temperature was set at 250 °C, with a split ratio of 20:1. Helium was used as the carrier gas at a constant flow rate of 24 mL/min. Compounds were identified by comparison of their mass spectra with those in the NIST and EPA library and confirmed by retention indices compared with literature data. On the other hand, HPLC–DAD elutions were carried out using a Waters 1525 system (equipped with a Waters 2998 detector and Kinetex C_18_ column, 150 mm, 5 μm) at a 1 mL/min flow rate using an H_2_O (0.1% formic acid)/acetonitrile mixture (90:10, *v*/*v*) for the first two minutes and a gradient to pure acetonitrile within the following 15 min.

PTX HPLC analysis was performed under isocratic conditions using a Chromsystems ODS column (25 µm, 125 × 4.6 mm), supplied by Chromsystems Instruments & Chemicals GmbH (Gräfelfing, Germany), and a HPLC system (Shimadzu, Tokyo, Japan); detection: UV-Vis detector (Shimadzu, Tokyo, Japan); λ: 227 nm; flow rate: 1.0 mL/min; mobile phase: acetonitrile/MilliQ water (50:50 *v*/*v*). A calibration curve (5–100 µg/mL range) with R^2^: 0.9999 was obtained. The standard deviation of intra- and inter-day precision at 3 concentrations (10, 20, and 50 µg/mL) was below 3%, with accuracy ranging from 97.0% to 103.0%.

### 2.3. Preparation of Nanoparticles (NPs)

#### 2.3.1. SLN Preparation

The ‘cold dilution of microemulsion’ method was used to prepare SLNs [29]. Prior to microemulsion (µE) formation, the selected water-miscible solvent (EA) and water were mutually saturated (referred to as EA_s_ and W_s_, respectively). The µE was then prepared by dissolving the lipid matrix in EA_s_ and then dispersing it in W_s_ with an appropriate mixture of surfactants, cosurfactants, and cosolvents to ensure system stability.

Rapid dilution of the resulting µE with a defined amount of unsaturated water induced SLN formation through precipitation, as the solvent was extracted from the dispersed phase into the continuous aqueous phase.

For drug loading, varying amounts of PTX or C_n_-HTyrCA were individually dissolved in 1 mL of the prepared µE. Each drug-containing µE was then diluted with unsaturated water to trigger SLN precipitation.

In the presence of PTX, a 3% *w*/*v* Pluronic^®^F-68 water solution was used to dilute the µEs and to obtain stable SLNs. As reported in a previous paper by Chirio et al. [29], Pluronic^®^F-68 was used to obtain small and non-aggregated SLNs.

When HA decoration was required, the positively charged surfactant, CTAB, was added.

Gel filtration (GF) was used to separate free drugs and/or non-incorporated ingredients from drug-loaded SLNs, using Sepharose^®^CL-B4, a cross-linked agarose material packed in a polypropylene column (10 mL volume, approximately 90 mm length × 18 mm outer diameter). A mobile phase buffer (phosphate-buffered saline) was used to wash the molecules through the column. HA-decorated SLNs were prepared, aiming at active targeting. By electrostatic interaction between HA and CTAB introduced in the µE formulation [21], HA decoration on the SLN surface was obtained.

Briefly, 1 mL of the drug-loaded SLN suspension was added dropwise to 5 mL of HA aqueous solution (0.05% *w*/*v*) under continuous 30 min stirring.

Unreacted HA was removed by gel centrifugation (GC) using a Sephadex^®^G-25 column.

#### 2.3.2. NLC Preparation

The hot melting homogenization method [21] was applied to prepare by using an Ultra Turrax^®^T25 homogenizer (Ika Labortechnik, Staufen, Germany).

The aqueous phase and the oil phase, containing both solid and liquid lipids, were separately heated to 60 °C before mixing and then homogenized by a high-shear homogenizer for 2 min at 6000 rpm and were further sonicated in continuous mode (40 kHz, 305 W, 20 °C, 10 min) to obtain NLCs with a narrow size distribution (polydispersion index-P.I. ≤0.300) using a high-intensity ultrasonic processor (Cole-Palmer Instruments, Vernon Hills, IL, USA).

To obtain NLCs, we prepared blank emulsions with sesame oil and a mixture of a fatty acid (myristic acid) and Compritol^®^888 ATO or Precirol^®^ATO5 as a solid lipid. A mixture of Tween^®^80 and Epikuron^®^200 as surfactants was used in emulsions with 1% w/w total lipid. Then NLC suspension was obtained by cooling the emulsion up to room temperature. NLC suspensions were then stored at 4 °C.

NLCs with the most suitable mean diameters were selected for the incorporation of increasing amounts of PTX or C_8_-HTyrCA in the lipophilic phase before the addition of water.

3% *w*/*v* Pluronic^®^F-68 was added in the external phase as a stabilizer in the presence of PTX to avoid NLC aggregation.

Free drugs and drug-loaded NLCs were separated by GF using a Sepharose^®^CL-B4 column.

To avoid the thermal degradation of C_n_-HTyrCA in the NLC preparation process, the solvent injection technique was also used. Briefly, different lipophilic components were completely dissolved in an ethanol solution of a surfactant mixture. The alcohol solution was then withdrawn with a syringe equipped with a needle and injected into the aqueous phase, consisting of an aqueous solution of a stabilizing polymer (PVA 9000 or Pluronic^®^F-68), while keeping the mixture under constant stirring. This mixture was then transferred to the ultrasonic ice bath for 20 min until nanocarrier precipitation was achieved.

In addition, an increasing amount of CTAB, as a positively charged surfactant, was added to obtain HA decoration.

In NLCs prepared with GMS and 15 mg of CTAB (NLC_8_-CTAB), C_8_-HTyrCA and C_10_-HTyrCA were added, and the resulting NLCs were then HA-decorated.

### 2.4. Characterization of NPs

#### 2.4.1. Particle Size and Zeta Potential Measurement

The particle size and Zeta potential of all batches of NP formulations were evaluated by dynamic light scattering (DLS) using a Zetasizer Nano ZS90 (Malvern Instruments, Worcestershire, UK). Prior to each measurement, NP suspensions were diluted with Milli-Q water. Analyses were conducted at 25 °C, and each sample was measured in triplicate. Results are presented as the average diameter with the corresponding standard error (S.E.) provided by the instrument in order to assess the precision and reliability of the measurements.

#### 2.4.2. Determination of EE%

The drug content in NP suspensions, following elution through GF and GC columns, was quantified using the HPLC method described in Section 2.2. The fraction recovered after GF and GC separation was considered to represent the drug fully entrapped within the NP core. The EE% was calculated as the ratio of the drug entrapped in NPs (post-GF concentration) to the total drug present in the NP formulation (pre-GF concentration), multiplied by 100. The recovery yield, expressed as a percentage, was determined by comparing the total drug detected in the NP preparation with the initial drug amount used during formulation.

### 2.5. Stability Studies of NPs

The stability of the NPs was evaluated by monitoring changes in mean particle size, Zeta potential, and EE% after 15-day storage at 4 °C.

### 2.6. In Vitro Drug Release

Release of C_8_-HTyrCA from SLNs and from NLCs was evaluated using the non-equilibrium dialysis method [30]. A multicompartmental rotating cell system was used; donor and receptor compartments had a volume of 1.5 mL. The experiments were carried out at room temperature. As a hydrophilic membrane, Servapor^®^ dialysis tubing (Serva, Heidelberg, Germany), with a cut-off of 12,000–14,000 Da, was used; the effective membrane area was 2 cm^2^. The receiving medium was a 1% *v*/*v* Tween^®^20 hydroalcoholic solution (ethanol:PBS 0.1 M Ph = 7.4 20:80 *v*/*v*). At fixed times, the receptor solution was tipped out, and the cell was refilled with fresh receiving medium. C_8_-HTyrCA concentration in the receptor phase was determined by the HPLC method. The release of the drug was monitored for 72 h. A saturated solution of C_8_-HTyrCA in 1% *v*/*v* Tween^®^20 hydroalcoholic solution (ethanol:PBS 0.1 M pH = 7.4, 20:80 *v*/*v*) was used as a reference.

### 2.7. In Vitro Studies

#### 2.7.1. Cell Lines

Human breast cancer MDA-MB-231 cells were purchased from the American Type Cell Collection (ATCC). MDA-MB-231 cells were generated by parental cells with a stepwise selection in a medium, increasing PTX concentration every 5 passages from 0 to 1 µM, and then maintained in the 1 µM PTX-containing medium. Cells were grown in DMEM medium with 1% *v*/*v* penicillin-streptomycin and 10% fetal bovine serum at 37 °C and 5% CO_2_. In each experimental set, cells were incubated with SLNs at a 0.5–5 µg/mL concentration range or with a solution of PTX at the same concentration as reference. Samples under study were as follows: free PTX or C_8_-TyrCA, SLN TL-PTX, SLN C_8_-HTyrCA, SLN TL-PTX-HA, SLN C_8_-HTyrCA-HA, SLN TL-PTX+ SLN C_8_-HTyrCA, SLN TL-PTX-HA+ SLN C_8_-HtyrCA, and SLN TL-PTX-HA + SLN C_8_-HTyrCA-HA.

#### 2.7.2. Viability

A total of 1 × 10^5^ cells were seeded into 96-well plates and incubated for 24 or 48 h with concentrations ranging from 0.5 to 5 µg/mL, as described in Section 2.7.1. Cell viability was assessed using the ATPLite assay (PerkinElmer, Waltham, MA, USA), a chemiluminescence-based assay. Viability in untreated cells was considered 100%; results were expressed as a percentage of viable cells toward untreated cells.

### 2.8. Statistical Analysis

All data entered in the text and reported in figures are provided as means ± standard deviation (S.D.). Data were analyzed using one-way ANOVA and Tukey’s test, with *p* < 0.05 considered statistically significant. Particle diameters and Zeta potential values are expressed as mean ± S.E., as reported by the instrument that operates with the Zetasizer Software 7.13 (Malvern Instruments, Worcestershire, UK).

## 3. Results and Discussion

In light of the inherent limitations of current chemotherapeutic strategies for TNBC, particularly resistance that undermines their long-term efficacy, we developed SLNs and NLCs individually loaded with PTX or a series of C_n_-HTyrCA (C_8_-, C_10_-, C_12_-HTyrCA) synthesized in our laboratories. As with many other chemotherapeutic agents, PTX—despite being a widely used mitotic inhibitor in TNBC treatment—is often rendered ineffective by the frequent occurrence of drug resistance, which contributes significantly to chemotherapy failure and disease relapse [31].

An extensive formulation phase was carried out to optimize the production parameters for SLNs and NLCs, with the aim of achieving high drug entrapment efficiency and long-term stability. The main objective was to obtain HA-decorated SLNs and NLCs for the co-administration of PTX and the most promising C_n_-HTyrCA and to evaluate their therapeutic potential in TNBC cell lines. The innovative aspect of this research lies in the development and in vitro evaluation of a novel therapeutic strategy based on the co-administration of SLNs and NLCs individually loaded with PTX and C_n_-HTyrCA. This approach allows for flexible dose modulation of each compound according to therapeutic needs while simultaneously addressing chemoresistance mechanisms in TNBC research.

### 3.1. Formulation Studies of NPs and NP Mean Diameters

#### 3.1.1. SLN Formulation

PTX-containing SLN: Different solid lipids (fatty acids, mono- and triglycerides) were chosen as SLN matrices to incorporate different amounts of PTX (Table 1). SLNs were also prepared using PTX and lecithin as the only components of the lipophilic matrix (named SLN-PTX). In this formulation, sodium taurocholate was added to obtain a transparent microemulsion able to form SLNs.

SLN TL-PTX containing 5 mg PTX were chosen to be HA-decorated, considering their relatively low mean diameter also in the presence of an intermediate PTX amount. The resulting mean diameter (±S.E.) of SLN TL-PTX-HA was 468.8 ± 16.8 with P.I. = 0.376. The HA decoration determined a marked increase in mean diameters of HA-decorated PTX-loaded SLNs, as already demonstrated in previous studies [21,32].

C_n_HTyrCA-containing SLN: With the aim of formulating C_n_-HyrCA-loaded SLNs with a similar composition to PTX-loaded ones, different amounts (2.5/5/10/15 mg) of C_8_-HTyrCA, or C_10_-HTyrCA, or C_12_-HTyrCA were loaded in SLNs with TL matrix (Table 2).

The average size of the SLNs did not vary in a significant way depending on the chain length of C_n_-HTyrCA. Furthermore, within the same set of SLNs, increasing amounts of the same C_n_-HTyrCA did not lead to significant variations in mean diameters; indeed, no correlation was found between the average diameters and the type or concentration of the C_n_-HTyrCA derivative. Even if the lipophilic derivative can acquire amphiphilic characteristics as the chain length increases, and, consequently, it would play a role in μE and in SLN stabilization, we were not able to demonstrate this assumption with significant experimental data.

The addition of CTAB does not alter mean diameters of SLNs (undecorated SLN), whereas decoration with HA has a significant impact on the size, doubling the initial dimensions (Table 3).

#### 3.1.2. NLC Formulations

*Hot homogenization and cooling method:* we formulated NLCs using the hot homogenization and cooling method as described in the previous work [21], and we evaluated the use of glyceryl distearate (Precirol^®^ATO5) versus glyceryl dibehenate (Compritol^®^888ATO) in the presence of myristic acid and sesame oil as the liquid lipid, since it had already been evaluated against other oils. Table 4 shows the qualitative and quantitative compositions of blank NLCs with the average diameters obtained.

The diameters did not change drastically using Precirol^®^ATO5 or Compritol^®^888ATO (C_18_ and C_22_ acyl chains, respectively); anyway, Precirol^®^ATO5 (NLC_b_) was selected to develop C_n_-HTyrCA- and PTX-loaded NLCs (Table 5). Among the three synthesized C_n_-HTyrCA, the octyl derivative was selected as the model to perform the early formulation studies.

In presence of PTX, the emulsion formulations were prepared with and without Pluronic^®^F-68, used as a stabilizer (NLC_b_-PL-PTX and NLC_b_-PTX, respectively), to determine any differences in the average diameter of the resulting NLCs. Moreover, increasing amounts of PTX were added in NLC_b_-PL.

The presence of Pluronic^®^F-68 in NLC_b_ containing PTX does not imply a consistent reduction in size. NLCs are probably sterically protected by the ethoxylated non-ionic surfactant (Tween^®^80), preventing them from aggregation. In contrast, increasing amounts of PTX lead to an increase in mean diameters, as also reported in the literature data [33], possibly due to the packaging in the lipid core.

*Solvent injection method*: to determine whether the high temperatures used to prepare NLCs could pose a problem for C_n_-HTyrCA, blank NLCs were also prepared using the solvent injection method that works at room temperature. Ethanol was used to dissolve the lipids and two different stabilizing polymers were also tested (Table 6).

Considering the date reported in Table 6, it is clear that NLCs with GMS as a lipid matrix have the smallest average diameters of the entire series. This characteristic makes them particularly suitable for our purposes. Therefore, to achieve a positively charged surface on the NLCs, increasing amounts (10–30 mg) of the cationic surfactant CTAB were incorporated into the formulation NLC_8_, containing 30 mg GMS as solid lipid, as it was that with the lowest mean diameter among the formulations shown in Table 6. Subsequently, the HA solution was reacted with the positively charged NLCs to obtain HA-decorated NLCs. Mean diameters of the HA-decorated empty NLCs are reported in Table 7. When the HA solution was added to the 30 mg CTAB-containing NLC_8,_ a visible precipitate was observed, and therefore mean diameters were not determined. As expected, the HA decoration determined on NLC_8_ a slight increase in mean diameters in 10 and 15 mg CTAB-containing NLCs.

C_8_-HTyrCA or C_10_-HTyrCA (5 mg) was introduced in the formulation containing 15 mg CTAB (NLC_8_-CTAB); the resulting NLCs (NLC_8_-C_8_-HTyrCA and NLC_8_-C_10_-HTyrCA) were also decorated with HA (NLC_8_-C_8_-HTyrCA-HA and NLC_8_-C_10_-HTyrCA-HA) as described in paragraph 2.3.1. In this case, the HA decoration determined a marked increase in mean diameters, which was more pronounced than that observed in empty NLCs (Table 8).

### 3.2. Zeta Potential of SLNs and NLCs

Zeta potential was measured for NPs (NLCs and SLNs) containing CTAB before and after HA decoration to confirm the successful decoration (Table 9).

The presence of CTAB in all the NP formulations imparts a positive surface charge, as confirmed by the Zeta potential value, which is always positive for all formulations containing CTAB. The interaction with the negative charges of HA induces the surface charge of the coated NPs to become highly negative, with the decoration being confirmed by a Zeta potential in the −11/−20 mV range.

SLN C_8_-HTyrCA and SLN C_10_-HTyrCA, containing 30 mg of CTAB, show a high Zeta potential value (around +30 mV), which provides stability to the system.

As for the NLC samples, NLC_8_ without CTAB and drug-free exhibit a negative Zeta potential, similar to the NLC_b_-PTX and NLC_b_-PL-PTX (3 mg). The incorporation of increasing amounts of CTAB into the NLC_8_ results in an increase in the positive absolute value of the Zeta potential. The inclusion of C_n_-HTyrCA slightly decreases the Zeta potential value, although the absolute value remains positive.

### 3.3. Entrapment Efficiency (EE%)

For those drug-loaded SLN formulations with favorable properties in terms of mean diameter and Zeta potential, the concentration of PTX and of C_n_-HTyrCA were quantified by HPLC for the determination of EE%.

Table 10 reports the PTX concentrations measured in the various SLN formulations, which were composed of different lipid matrices.

The highest PTX EE% is obtained in SLNs containing TL as a lipid matrix. Moreover, among all the 3 mg PTX-containing formulations (SLN S2-PTX, SLN P2-PTX, SLN-GMS-PTX3, and SLN TL-PTX), SLN TL-PTX exhibits an EE% of 62%, compared to 47% for SLN S2-PTX, 35% for SLN P2-PTX, and 51% for SLN GMS-PTX3. When comparing 10 mg PTX-containing formulations, SLN TL-PTX shows a consistently higher EE% than SLN-PTX.

In SLNs with GMS as a lipid matrix, even tripling the amount of PTX does not result in an increase in EE%, whereas, if the amount of GMS is doubled, a significant decrease in EE% occurs. This reduction is likely due to the difficulty of incorporating PTX within the matrix.

In SLN TL-PTX, increasing the PTX content initially increases the EE%, but this is followed by a significant decrease, which can be explained by matrix saturation.

Table 11 presents the EE% of the various C_n_-HTyrCA (C_8_-HtyrCA, C_10_-HtyrCA and C_12_-HTyrCA) incorporated into the TL matrix at increasing concentrations.

**Figure 1:**
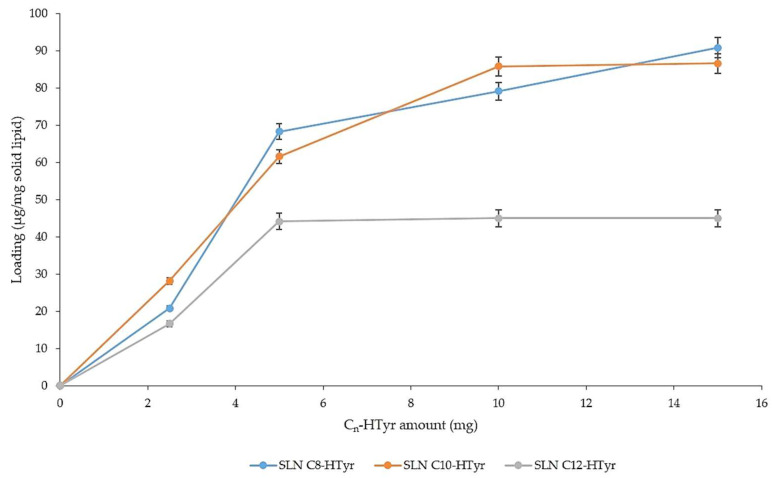
Loading (µg C_n_-HTyrCA/mg solid lipid) of C_n_-HTyrCA as a function of the amount introduced into the starting µE.

As the amount of C_n_-HTyrCA incorporated into the formulation increases, the loading (intended as µg of C_n_-HTyrCAmg of lipid matrix) increases until it reaches a plateau value, probably due to the lipid matrix saturation that occurred corresponding to 5 mg of each C_n_-HTyrCA (Figure 1). The plateau value was quite similar for C_8_-HTyrCA and C_10_-HTyrCA but was almost halved for C_12_-HTyrCA, indicating a lower affinity for the lipid matrix.

Considering the overall results, SLNs with 5 mg of C_8_-HTyrCA and C_10_-HTyrCA were chosen to be HA-decorated, as no further EE% increase was noted by increasing their amount in the formulations (Table 11).

In the formulation containing 5 mg of C_8_-HTyrCA and C_10_-HTyrCA, 30 mg of CTAB was added, and the EE% was calculated to be 88 and 84%, respectively. The formulation with C_12_-HTyrCA was not prepared, as its EE% (Table 11) was always lower than those obtained for C_8_-HTyrCA and C_10_-HTyrCA at the corresponding concentrations.

The presence of CTAB minimally influences the EE% of C_n_-HTyrCA in SLNs.

C_n_-HTyrCA concentration in HA-decorated SLNs after GC was equal to 1/6 of the concentration after GF. SLNs were then concentrated to the original concentration under nitrogen flow.

In Table 12, EE% of NLCs prepared with different techniques is reported.

Regardless of the NPs prepared, the EE% in NLCs is significantly lower than in SLNs. NLCs containing PTX prepared using the hot method showed EE% values in the 45–53% range, with an acceptable recovery. NLC_b_ with and without Pluronic^®^F68 are analyzed with the same amount of PTX (3 mg) to evaluate the capacity of Pluronic^®^F68 to increase the PTX entrapment. The presence of Pluronic^®^F68 slightly improved the PTX EE% of NLCs (comparison between NLC_b_-PTX and NLC_b_-PL-PTX 3 mg). NLCs containing C_8_-HTyrCA were prepared by both hot and cold methods for comparison. Without the application of heat, there was an increase in EE% in the NLC_8_-C_8_-HTyrCA formulation. The yield remained low for the hot method, with a recovery of 30%. In NLCs prepared with the cold method, both recovery and EE% of C_10_-HTyrCA were even lower than C_8_-HTyrCA. For this reason, the incorporation test for the C_12_-HTyrCA was not performed.

### 3.4. Release Studies

**Figure 2:**
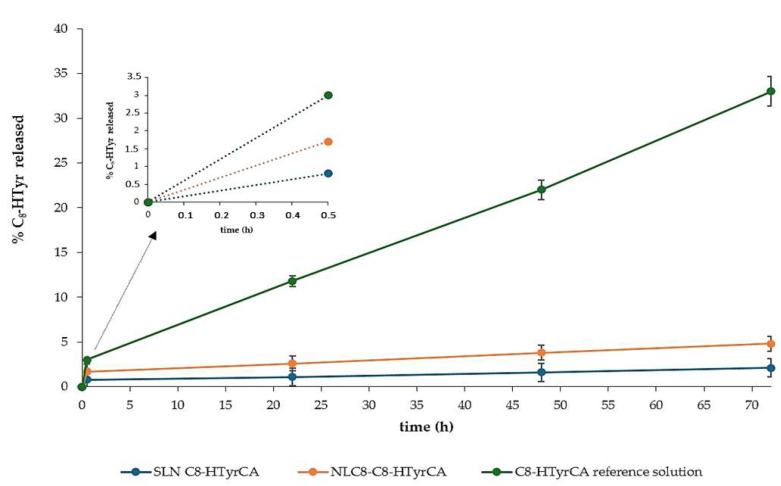
Release profile of C_8_-HTyrCA from SLN and NLC compared to reference solution with the detail of the initial 30-min release.

In Figure 2, the release profile of C_8_-HTyrCA from SLNs and NLCs, prepared with the solvent injection method, is shown and compared to its aqueous solution. The release studies were conducted only on C_8_-HTyrCA, as no significant differences were observed between the C_8_ and C_10_ derivatives in terms of EE% in SLNs. For NLCs prepared under cold conditions, C_8_-HTyrCA exhibited a higher EE% compared to C_10_-HTyrCA. Furthermore, the release studies were primarily performed to assess the influence on release patterns of the different lipid matrix configurations in SLNs and NLCs.

After 72 h, the percentage of C_8_-HTyrCA released from SLNs and NLCs was less than 5%, compared to 33% from the solution. These findings suggest that the active molecule is effectively entrapped within the lipid matrix and is gradually released over time.

NLCs and SLNs exhibit the same release profile, but NLCs show an initial burst release, probably due to surface-located and/or adsorbed C_8_-HTyrCA.

### 3.5. Stability Studies

Stability studies were conducted over time in terms of mean size and Zeta potential of both non-decorated (post-GF) and HA-decorated (post-GC) NP systems in order to evaluate how the systems (NLCs and SLNs) maintain their original parameters over the duration of the study (15 days).

SLNs are more stable in terms of mean diameter than NLCs (Table 13), probably due to a more pronounced rigidity of the lipid matrix. The lower rigidity of the lipid matrix in NLCs could also confirm the above mentioned burst effect in release studies.

For NLCs, there is a progressive increase in mean diameters over 15 days, which is greater for the undecorated NLC_8_ (post-GF) than for the decorated ones (post-GC). The undecorated SLNs show unchanged size over the 15 days of observation, with a slight increase observed in those HA-decorated.

For the undecorated NLCs, slight fluctuations in Zeta potential are observed. As for the negatively charged decorated ones, after 15 days, a decrease of about 50% in the Zeta potential value is seen it could indicate an unstable decoration.

HA decoration appears to be more stable on the SLN surface, as the Zeta potential value remains almost constant after 15 days.

Since SLNs proved to be more stable than NLCs over the 15-day observation, stability tests of PTX-loaded systems were only conducted on TL-SLNs (Table 14).

The trend of the diameters and Zeta potentials is similar to that observed for SLNs containing C_8_-HTyrCA, especially for the decorated SLNs (post-GC), which increase their diameter by almost 30% over 15 days. Zeta potential of both decorated (post-GC) and non-decorated (post-GF) decreases slightly (about 13%).

### 3.6. In Vitro Test

**Figure 3:**
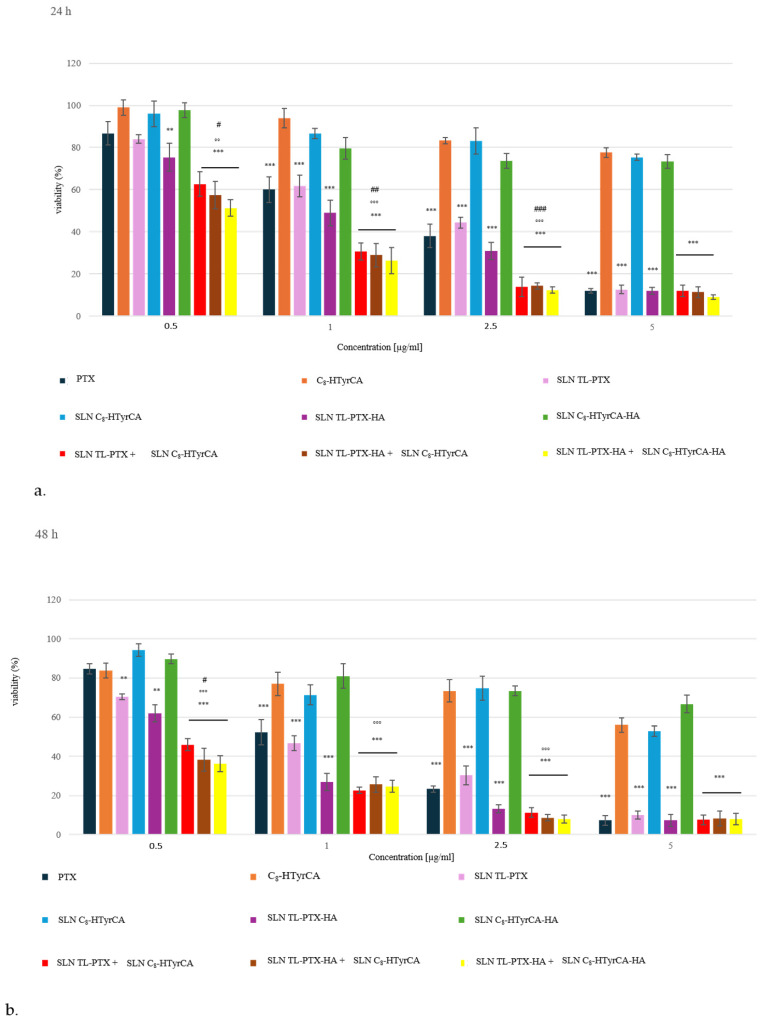
Cell viability of MDA-MB-231 cells incubated with the indicated concentrations of free PTX, C_8_-HTyrCA, or their SLN formulations, with or without HA, at 24 h (**a**) and 48 h (**b**). Results are mean ± SD (n = 3 experiments), in quadruplicates. ** *p* < 0.01, *** *p* < 0.001: vs. untreated cells, considered 100%; °° *p* < 0.01, °°° *p* < 0.001: vs. PTX; ^#^ *p* < 0.05, ^##^ *p* < 0.01, ^###^ *p* < 0.001: vs. SLN PTX or SLN PTX-HA.

MDA-MB-231 cells showed a dose- and time-dependent decrease in cell viability with PTX at each concentration and at each time point (Figure 3). The loading of PTX in SLNs progressively increased its anti-tumor efficacy, making PTX significantly cytotoxic at 1 µg/mL after 24 h or even at 0.5 µg/mL after 48 h. Except for the 5 µg/mL PTX concentration (the highest one), the cytotoxic effect seemed to be greater when PTX was carried by SLN TL-PTX-HA, suggesting that HA actively targeted the SLN to the cancer cells, favoring the drug delivery and subsequent cytotoxicity; this effect was more pronounced at 48 h than at 24 h. On the other hand, free C_8_-HTyrCA, SLN C_8_-HTyrCA and SLN C_8_-HTyrCA-HA were not toxic at each concentration and time point. SLN C_8_-HTyrCA, however, increased the cytotoxicity of SLN TL-PTX or SLN TL-PTX-HA compared to PTX in the concentration range 0–5–1–2.5 µg/mL, mainly at 24 h. The best cytotoxicity was achieved by the combination of SLN C_8_-HTyrCA-HA and SLN TL-PTX-HA that was superior to PTX, SLN TL-PTX or SLN TL-PTX-HA in the low concentration ranges (0.5–2.5 µg/mL) at 24 h. The difference between SLN C_8_-HTyrCA + SLN TL-PTX combinations, with or without HA, compared to SLN TL-PTX, or SLN TL-PTX-HA, was lower at 48 h, suggesting that the presence of C_8_-HTyrCA allows a faster cytotoxic effect of PTX, more evident at earlier time points. Given the significant toxicity induced by free PTX at 5 µg/mL, we did not observe a significant advantage exerted by SLN TL-PTX/SLN TL-PTX-HA alone or combined with C_8_-HTyrCA formulations at such high concentrations.

Overall, these results suggest that the co-incubation of HA-decorated or undecorated SLN TL-PTX with SLN C_8_-HTyrCA allows a better delivery of SLN into cancer cells; anyway, the positive effect of HA decoration, which was noted for SLN TL-PTX and SNL TL-PTX-HA, was not so evident in this case, as probably the synergistic action of C_8_-HTyrCA-loaded SLNs diminishes and hides the effect of HA. Further investigation will be needed to confirm this hypothesis.

Generally, the major advantages were obtained with low concentrations of PTX, indicating a potential employment of this strategy to lower the dosage of PTX required to exert cytotoxic effects. This means an increased anti-tumor effect with lower undesired adverse reactions.

## 4. Conclusions

The clinical use of natural compounds that modulate hypoxia-induced multidrug resistance is limited by poor solubility, instability, and low bioavailability. To address these challenges, we have developed HA-decorated lipid-based nanoparticles for the co-delivery of PTX and lipophilic HTyrCA esters. This study presents the rational design, development, and thorough physicochemical and biological characterization of HA-decorated SLNs and NLCs for the co-delivery of PTX and C_n_-HTyrCA, establishing them as an innovative nanotherapeutic platform against TNBC. The SLN-based formulations demonstrated superior drug entrapment, colloidal stability, and HA functionalization compared to the NLCs, with C_8_-HTyrCA showing the most favorable incorporation profiles within TL-based SLNs, exhibiting optimal loading capacity and formulation stability. HA decoration resulted in an increase in particle size and a shift towards a more negative Zeta potential, confirming effective conjugation and suggesting enhanced colloidal stability and the potential for CD44-mediated cellular uptake. Significantly, PTX-loaded SLNs exhibited enhanced antiproliferative effects against MDA-MB-231 cells at lower drug concentrations and at earlier time points, particularly with HA-mediated active targeting. Co-administering PTX and C_8_-HTyrCA in SLNs produced a synergistic cytotoxic response, suggesting their potential to reduce systemic toxicity and overcome resistance in TNBC. These findings highlight the potential of TL-based, HA-decorated SLNs as a nanoplatform for combination chemotherapy, which requires further validation in advanced biological models.

## Figures and Tables

**Table 1:** Quali-quantitative composition, mean diameters [nm] ± standard error (S.E.), and polydispersion index (P.I.) of SLN containing different amounts of PTX.

Ingredients	SLN S2-PTX	SLN P2-PTX	SLN TL-PTX				SLN GMS-PTX1	SLN GMS2-PTX1	SLN GMS-PTX3	SLN-PTX
Stearic acid	20 mg	-	-				-	-		-
Palmitic acid	-	20 mg	-				-	-		-
TL	-	-	50 mg				-	-		-
GMS	-	-	-				30 mg	60 mg	30 mg	-
EA_s_	200 µL	200 µL	200 µL				200 µL	200 µL	200 µL	200 µL
Epikuron^®^200	150 mg	150 mg	150 mg				150 mg	150 mg	150 mg	190 mg
Cremophor^®^RH60	50 µL	50 µL	50 µL				50 µL	50 µL	50 µL	100 µL
CTAB	30 mg	30 mg	30 mg				30 mg	30 mg	30 mg	30 mg
Benzyl alcohol	100 µL	100 µL	-				100 µL	100 µL	100 µL	-
PG	150 µL	100 µL	100 µL				100 µL	100 µL	100 µL	100 µL
H_2_O_s_	700 µL	700 µL	700 µL				700 µL	700 µL	700 µL	700 µL
Sodium taurocholate	-	-	-				-	-	-	20 mg
PTX	3 mg		3 mg	5 mg	7 mg	10 mg	1 mg		3 mg	10 mg
Pluronic^®^F-68 3% *w/v* water solution	3 mL									
Mean diameter [nm] ± S.E. (PI)	230.1 ± 4.4 (0.102)	249.9 ± 3.7 (0.152)	263.0 ± 3.0 (0.257)	183.4 ± 1.6 (0.216)	256.0 ± 2.3 (0.102)	203.4 ± 4.2 (0.250)	242.6 ± 3.0 (0.151)	171.8 ± 1.8 (0.189)	267.8 ± 3.7 (0.161)	209.0 ± 2.1 (0.159)

**Table 2:** Quali-quantitative composition, mean diameters [nm] ± S.E. (P.I.) of SLN containing increasing amounts of C_8_-HTyrCA, C_10_-HTyrCA, and C_12_-HTyrCA.

Ingredients	SLN C_n_-HTyrCA		
TL	60 mg		
EA_s_	200 µL		
Epikuron^®^200	150 mg		
Cremophor^®^RH60	50 µL		
H_2_O_s_	700 µL		
PG	100 µL		
H_2_O MilliQ	4 mL		
C_n_-HTyrCA	C_8_-HTyrCA	C_10_-HTyrCA	C_12_-HTyrCA
	Mean diameter [nm] ± S.E. (P.I.)		
2.5 mg	227.4 ± 1.6 (0.144)	232.3 ± 3.0 (0.159)	234.6 ± 2.6 (0.221)
5 mg	229.3 ± 2.0 (0.238)	237.0 ± 3.2 (0.272)	232.7 ± 3.0 (0.187)
10 mg	243.3 ± 2.9 (0.223)	219.0 ± 1.0 (0.229)	233.9 ± 4.1 (0.201)
15 mg	251.2 ± 3.4 (0.233)	198.9 ± 2.9 (0.260)	223.4 ± 4.7 (0.283)

**Table 3:** Mean diameters [nm] ± S.E. (P.I.) of SLN containing CTAB (30 mg) and C_8_-HTyrCA or C_10_-HTyrCA (5 mg).

SLN Samples	SLN C_8_-HTyrCA Mean Diameter [nm] ± S.E. (P.I.)	SLN C_10_-HTyrCA Mean Diameter [nm] ± S.E. (P.I.)
Undecorated SLN	228.5 ± 3.4 (0.281)	244.9 ± 0.9 (0.247)
HA decorated SLN	494.3 ± 10.0 (0.349)	400.2 ± 11.0 (0.308)

**Table 4:** Quali-quantitative composition and mean diameter [nm] ± S.E. (P.I.) of blank NLC prepared by hot homogenization method.

Ingredients	NLC_a_	NLC_b_
Myristic acid	75.0 mg	62.0 mg
Compritol^®^888ATO	17.5 mg	-
Precirol^®^ATO5	-	25.0 mg
Sesame oil	7.5 μL	7.5 μL
Tween^®^80	140.0 μL	150.0 μL
Epikuron^®^200	30.0 mg	30.0 mg
MilliQ water	9.8 g	9.8 g
Mean diameter [nm] ± S.E. (P.I.)	280.5 ± 7.3 (0.209)	250.2 ± 5.2 (0.182)

**Table 5:** Quali-quantitative composition and mean diameters [nm] ± S.E. (P.I.) of NLC containing PTX or C_8_-HTyrCA prepared by homogenization and cooling method.

Ingredients	NLC_b_-PTX	NLC_b_-PL-PTX			NLC_b_-C_8_-HTyrCA
Myristic acid	62.0 mg	62.0 mg			62.0 mg
Precirol^®^ATO5	25.0 mg	25.0 mg			25.0 mg
Sesame oil	7.5 μL	7.5 μL			7.5 μL
Tween^®^80	150.0 μL	150.0 μL			150.0 μL
Epikuron^®^200	30.0 mg	30.0 mg			30.0 mg
MilliQ water	9.8 g	9.8 g			9.8 g
Pluronic^®^F-68	-	0.3 g			-
C_8_-HTyrCA	-	-			5.0 mg
PTX	3.0 mg	1.5 mg	3.0 mg	5.0 mg	-
Mean diameter [nm] ± S.E. (P.I.)	281.3 ± 1.9 (0.285)	263.7 ± 1.3 (0.219)	283.7 ± 2.7 (0.192)	355.7 ± 3.2 (0.215)	272.3 ± 4.7 (0.237)

**Table 6:** Quali-quantitative composition [nm] ± S.E. (P.I.) of blank NLC prepared by solvent injection method.

Ingredients	NLC_1_	NLC_2_	NLC_3_	NLC_4_	NLC_5_	NLC_6_	NLC_7_	NLC_8_	NLC_9_
Trilaurin	15 mg	15 mg	15 mg	15 mg	15 mg	-	-	-	-
Myristic acid	-	-	-	**-**	**-**	15 mg	-	-	-
GMS	-	-	-	-	-	-	20 mg	30 mg	50 mg
Epikuron^®^200	30 mg	30 mg	50 mg	30 mg	30 mg	30 mg	30 mg	30 mg	30 mg
Compritol^®^ATO888	-	18 mg	18 mg	18 mg	18 mg	18 mg	-	-	-
Precirol^®^ATO5	18 mg	-	-	-	-	-	-	-	-
Sesame oil	10 μL	10 μL	10 μL	10 μL	10 μL	10 μL	15 µL	15 µL	15 µL
Ethanol	1 mL	1 mL	1 mL	1 mL	1 mL	1 mL	1 mL	1.5 mL	1.5 mL
PVA 9000	10 mg	10 mg	10 mg	100 mg	-	10 mg	10 mg	5 mg	5 mg
Pluronic^®^F-68	-	-	-	-	10 mg	-	-	-	-
MilliQ water	10 mL	10 mL	10 mL	10 mL	10 mL	10 mL	10 mL	5 mL	5 mL
Mean diameter [nm] ± S.E. (P.I.)	264.8 ± 2.3 (0.197)	254.9 ± 2.0 (0.189)	315.9 ± 1.9 (0.199)	586.7 ± 4.7 (0.225)	289.8 ± 4.1 (0.202)	295.5 ± 2.9 (0.230)	205.9 ± 2.8 (0.179)	199.5 ± 3.5 (0.173)	240.4 ± 1.7 (0.187)

**Table 7:** Mean diameters [nm] ± S.E. (P.I.) of blank NLC_8_ containing increasing amounts of CTAB and prepared by solvent injection method.

CTAB Amount (mg)	NLC_8_-CTAB Mean Diameter [nm] ± S.E. (P.I.)	NLC_8_-HA Mean Diameter [nm] ± S.E. (P.I.)
10	116.7 ± 2.1 (0.134)	123.9 ± 5.5 (0.178)
15	168.1 ± 6.2 (0.167)	186.9 ± 28.4 (0.189)
30	111.1 ± 2.5 (0.132)	nd*

nd* = not determined.

**Table 8:** Mean diameter [nm] ± S.E. (P.I.) of NLC_8_-C_n_-HTyrCA undecorated and decorated with HA prepared with solvent injection method.

NLC Samples	Mean Diameter [nm] ± S.E. (P.I.)
NLC_8_-C_8_-HTyrCA	111.9 ± 6.1 (0.162)
NLC_8_-C_8_-HTyrCA-HA	390.1 ± 19.7 (0.195)
NLC_8_-C_10_-HTyrCA	186.9 ± 19.7 (0.182)
NLC_8_-C_10_-HTyrCA-HA	510.2 ± 10.7 (0.235)

**Table 9:** Zeta potential [mV] ± S.E. of decorated and undecorated NPs.

NP Samples	Zeta Potential [mV] ± S.E.
SLN TL-PTX	+32.8 ± 3.3
SLN TL-PTX-HA	−15.5 ± 2.1
SLN C_8_-HTyrCA	+28.6 ± 2.3
SLN C_8_-HTyrCA-HA	−15.3 ± 2.8
SLN C_10_-HTyrCA	+32.6 ± 1.9
SLN C_10_-HTyrCA-HA	−11.8 ± 4.4
NLC_8_	−25.4 ± 2.9
NLC_8_-CTAB (10 mg)	+5.7 ± 3.2
NLC_8_-CTAB (15 mg)	+25.5 ± 6.5
NLC_8_-HA (15 mg)	−16.5 ± 4.1
NLC_8_-C_8_-HTyrCA	+13.7 ± 2.2
NLC_8_-C_8_-HTyrCA-HA	−20.1 ± 1.7
NLC_8_-C_10_-HTyrCA	+15.9 ± 4.2
NLC_8_-C_10_-HTyrCA-HA	−15.2 ± 2.5
NLC_b_-PTX	−22.4 ± 2.9
NLC_b_-PL-PTX (3 mg)	−24.2 ± 3.2

**Table 10:** PTX amount added, PTX concentration (mg/mL) before and after GF (pre- and post-GF, respectively) and EE% in different SLN formulations.

SLN Samples	PTX (mg)	PTX Concentration Pre-GF (mg/mL)	PTX Concentration Post-GF (mg/mL)	EE%
SLN S2-PTX	3	0.710	0.334	47
SLN P2-PTX	3	0.720	0.252	35
SLN-GMS-PTX1	1	0.230	0.125	54
SLN-GMS-PTX3	3	0.690	0.355	51
SLN-GMS2-PTX1	1	0.230	0.047	20
SLN-PTX	10	2.400	0.503	21
SLN TL-PTX	3	0.690	0.430	62
	5	1.057	0.771	73
	7	1.630	0.636	39
	10	2.430	0.680	28

**Table 11:** C_n_HTyrCA amount, concentration (mg/mL) before and after GF (pre- and post-GF) and EE% of different SLN formulations (SLN C_8_-HTyrCA; SLN C_10_-HTyrCA; SLN C_12_-HTyrCA).

C_n_HTyrCA	Amount Added (mg)	Concentration in SLN Pre-GF (mg/mL)	Concentration in SLN Post-GF (mg/mL)	EE%
C_8_-HTyrCA	2.5	0.40	0.25	63
	5	0.90	0.82	91
	10	1.20	0.95	79
	15	1.50	1.09	73
C_10_-HTyrCA	2.5	0.38	0.35	92
	5	0.80	0.74	92
	10	1.10	1.03	94
	15	1.30	1.04	80
C_12_-HTyrCA	2.5	0.35	0.20	57
	5	0.70	0.53	76
	10	0.90	0.54	60
	15	1.10	0.54	49

**Table 12:** Concentration (mg/mL) of PTX and C_n_-HTyrCA before and after GF (pre- and post-GF) and EE% in NLCs prepared with different techniques (hot homogenization and solvent injection).

NLC Sample	Preparation Method	Active Compound Concentration		EE%
		Pre-GF (mg/mL)	Post-GF (mg/mL)	
NLC_b_-PTX	Hot homogenization	0.22	0.10	45
NLC_b_-PL-PTX (3 mg)		0.30	0.16	53
NLC_b_-C_8_-HTyrCA		0.15	0.03	20
NLC_8_-C_8_-HTyrCA	Solvent injection	0.39	0.14	36
NLC_8_-C_10_-HTyrCA		0.34	0.06	18

**Table 13:** Mean diameter [nm] and Zeta potential [mV] ± S.E. overtime (15 days) of C_8_-HTyrCA in NLC and SLN non-decorated (post-GF) and HA-decorated (post-GC).

Time	NLC_8_-C_8_-HTyrCA				SLN-C_8_-HTyrCA			
	Mean Diameter [nm] ± S.E.		Zeta Potential [mV] ± S.E.		Mean Diameter [nm] ± S.E		Zeta Potential [mV] ± S.E.	
	Post-GF	Post-GC	Post-GF	Post-GC	Post-GF	Post-GC	Post-GF	Post-GC
t_0_	111.9 ± 6.1	390.1 ± 19.7	+ 13.7 ± 2.2	−20.1 ± 1.7	181.8 ± 1.1	482.9 ± 29.6	+12.1 ± 1.5	−5.2 ± 0.6
t_48h_	146.7 ± 2.8	409.6 ± 35.9	+ 14.9 ± 2.4	−14.7 ± 1.0	164.5 ± 2.0	646.3 ± 23.3	+13.7 ± 1.0	−7.5 ± 1.4
t_7dd_	276.6 ± 5.2	523.5 ± 38.5	+ 16.7 ± 2.2	−15.8 ± 1.9	192.0 ± 3.7	643.4 ± 26.3	+13.3 ± 0.7	−6.1 ± 0.9
t_15dd_	761.9 ± 10.5	836.0 ± 160.6	+ 11.3 ± 1.7	−9.1 ± 1.2	179.5 ± 1.9	629.2 ± 18.5	+12.2 ± 1.1	−6.2 ± 0.1

**Table 14:** Mean diameter [nm] and Zeta potential [mV] ± S.E. over time (15 days) of SLN TL-PTX non-decorated (post–GF) and HA-decorated (post-GC).

Time	SLN TL-PTX			
	Mean Diameter [nm] ± S.E.		Zeta Potential [mV] ± S.E.	
	Post-GF	Post-GC	Post-GF	Post-GC
t_0_	183.4 ± 1.6	468.8 ± 16.8	+32.7 ± 3.3	−15.5 ± 2.1
t_48h_	213.2 ± 5.6	501.8 ± 20.1	+30.1 ± 2.2	−15.3 ± 1.2
t_7dd_	223.4 ± 2.9	510.6 ± 20.1	+30.0 ± 2.4	−14.9 ± 1.2
t_15dd_	235.1 ± 1.2	606.3 ± 22.6	+28.7 ± 2.3	−13.4 ± 2.1

## Data Availability

The original contributions presented in this study are included in the article. Further inquiries can be directed to the corresponding author(s).

## Abbreviations

The following abbreviations are used in this manuscript:

µE	Microemulsion
AF	Aminoflavone
C_10_-HTyrCA	Hydroxytyrosolcarboxylic acid decyl ester
C_12_-HTyrCA	Hydroxytyrosolcarboxylic acid dodecyl ester
C_8_-HTyrCA	Hydroxytyrosolcarboxylic acid octyl ester
C_n_-HTyrCA	Hydroxytyrosolcarboxylic acid esters
CTAB	Cetyltrimethylammonium bromide
CUR	Curcumin
DMSO	Dimethyl sulfoxide
DOXO	Doxorubicin
EA	Ethyl acetate
EA_s_	Water saturated ethyl acetate
EE%	Entrapment efficiency percentage
GC	Gel centrifugation
GF	Gel filtration
GMS	Glyceryl monostearate
HA	Hyaluronic acid
HER 2	Human Epidermal Growth Factor Receptor 2
HIF-1	Hypoxia-inducible factor-1
HTyr	7-hydroxytyrosol
HTyrCA	Hydroxytyrosolcarboxylic acid
MDR	Multidrug resistance
NLCs	Nanostructured lipid carriers
NPs	Nanoparticles
P.I.	Polydispersion index
PG	Propylene glycol
P-gp	P-glycoprotein
PTX	Paclitaxel
SLNs	Solid lipid nanoparticles
TL	Trilaurin
TNBC	Triple negative breast cancer
W_s_	EA saturated water
